# AICD: an integrated anti-inflammatory compounds database for drug discovery

**DOI:** 10.1038/s41598-019-44227-x

**Published:** 2019-05-23

**Authors:** Kun Wang, Jianyong Xiao, Xiaodong Liu, Zhuqiao Jiang, Yujuan Zhan, Ting Yin, Lina He, Fangyuan Zhang, Shangping Xing, Bonan Chen, Yingshi Li, Fengxue Zhang, Zaoyuan Kuang, Biaoyan Du, Jiangyong Gu

**Affiliations:** 1 Research Center of Integrative Medicine, School of Basic Medical Science, Guangzhou University of Chinese Medicine, Guangzhou, 510006 China; 2 Department of Pathology, Guangzhou University of Chinese Medicine, Guangzhou, 510006 China; 3 Department of Biochemistry, Guangzhou University of Chinese Medicine, Guangzhou, 510006 China; 4 Department of Anaesthesia and Intensive Care, The Chinese University of Hong Kong, Hong Kong, 999077 China; 5 The Second Clinical College, Guangzhou University of Chinese Medicine, Guangzhou, 510006 China

**Keywords:** Computational biology and bioinformatics, Drug discovery

## Abstract

Systemic or local inflammation drives the pathogenesis of various human diseases. Small compounds with anti-inflammatory properties hold great potential for clinical translation. Over recent decades, many compounds have been screened for their action against inflammation-related targets. Databases that integrate the physicochemical properties and bioassay results of these compounds are lacking. We created an “Anti-Inflammatory Compounds Database” (AICD) to deposit compounds with potential anti-inflammation activities. A total of 232 inflammation-related targets were recruited by the AICD. Gene set enrichment analysis showed that these targets were involved in various human diseases. Bioassays of these targets were collected from open-access databases and adopted to extract 79,781 small molecules with information on chemical properties, candidate targets, bioassay models and bioassay results. Principal component analysis demonstrated that these deposited compounds were closely related to US Food and Drug Administration-approved drugs with respect to chemical space and chemical properties. Finally, pathway-based screening for drug combination/multi-target drugs provided a case study for drug discovery using the AICD. The AICD focuses on inflammation-related drug targets and contains substantial candidate compounds with high chemical diversity and good drug-like properties. It could be serviced for the discovery of anti-inflammatory medicines and can be accessed freely at http://956023.ichengyun.net/AICD/index.php.

## Introduction

Inflammation is initiated in response to tissue damage by pathogens^1^. In general, inflammation is believed to provide protection for the body, but an exaggerated and persistent inflammatory response is detrimental to human health^2^. In fact, dysregulated inflammation has been identified in the pathogenesis of almost all types of human diseases or symptoms. For example, overactivation of tissue-resident or infiltrating macrophages can cause secondary injury to the spinal cord, lungs and liver^3,4^. This secondary injury can lead to irreversible and often more serious lesions to organs (e.g., fibrosis and cirrhosis)^5^. Persistent local inflammation has also been revealed to have critical roles in cancer development^6^.

Conversely, medications that target inflammation have been applied widely for the control of diseases or symptoms. Non-steroidal anti-inflammatory drugs, corticosteroids and disease-modifying antirheumatic drugs can suppress innate or adaptive inflammatory responses dramatically. These medications, therefore, are adopted frequently for treatment of arthritis^7^. Regular intake of the selective inhibitor of cyclooxygenase (COX)-2, celecoxib, which can attenuate chronic inflammatory responses, has shown promise in prevention of colon-cancer development^8^. Despite considerable success, application of anti-inflammatory medications may be restrained due to serious side effects or insufficient efficacies against particular diseases. Accordingly, drug screening aiming to identify novel anti-inflammatory compounds remains a highly active research topic.

Molecules used as active substances can be divided into two classes: “small” and “large”. A small molecule is a low-molecular-weight compound that can regulate a biological process, and most drugs are small molecules^9^. Small molecules have been the focus of drug discovery in academia and the pharmaceutical industry. Small-molecule drugs have been used for treatment of inflammation-associated disorders, including chronic musculoskeletal degenerative diseases (gefitinib and imatinib)^10^, neuroinflammation (aspirin)^11^ and inflammatory bowel disease^12^.

There are several inflammation-related databases, such as Infevers^13^ (which focuses on mutations of the genes associated with autoimmune disease), IBDSite^14^ (inflammatory bowel disease), KERIS^15^ (gene responses to inflammation between species). However, a database that integrates small molecules with anti-inflammatory activities is not available.

In the present study, we integrated all relevant biological information of small molecules against inflammation to construct an “anti-inflammatory compounds database” (AICD). The AICD includes small molecule-related information, such as physicochemical properties, as well as the biological activity of small molecules against inflammation-related targets and experimental models.

## Construction and Content

### Data collection and deployment onto the Internet

Four types of data, *Targets*, *Molecules*, *Bioassay result*s and *Models*, were included in the AICD.

#### Targets

Firstly, we extracted inflammation-related protein targets from literatures manually (Table a in Additional File 1). In the process of targets collection, curator 1 and curator 2 collected the targets independently; then curator 3 made a final decision. The judgements of curators were documented in the supplementary information (Table b in Additional File 1) based on three separated tables, i.e. table of conclusions from curator 1, 2, and 3 (data not shown). When agreement for one particular target was achieved by curator 1 and 2, curator 3 selected one of the relevant references to browse and make a quick decision. In situations that curator 1 and 2 were not in agreement (e.g. Table b Beta-1,4-galactosyltransferase 5), curator 3 inquired about functional studies of the particular target and made a final decision. The targets list (153 targets) and PubMed IDs of related publications were put into the supplementary information (Table a in Additional File 1). Secondly, 117 inflammation-related proteins (Additional File 2) which were targets of anti-inflammatory drugs were obtained from DrugBank^16^ and Therapeutic Target Database (TTD)^17^. Then targets from different sources were merged to generate a target list (232 targets) without duplicates. Of these 232 targets, 25 targets were not assessed for activity assay. The targets (207) with potential bioactive small-molecules were listed in the Supplementary Dataset 1. To include as many as druggable inflammatory targets, all 232 targets were collected into the AICD. We will update AICD in case the bioactivity assay is available for these 25 targets in the future. For each target, the gene symbol, gene ID, gene name and UniProt ID were included in the list of *Targets*. Users can find target information through the link to the UniProt database. The final list can be found in the *Browse*/*Targets* section of the AICD website.

#### Molecules and bioassay results

Small-molecule compounds which were tested in the bioassays of the documented inflammatory targets were collected from several open-access sources: PubChem, BindingDB^18^, ChEMBL^19^ and ZINC^20^. Therefore, two types of data, *Molecules* and their *Bioassay results*, were generated. The data *Molecules* consisted of Compound Identifier (CID), Chemical Abstracts Service (CAS) Number and molecular name, which were extracted from PubChem, BindingDB^18^, ZINC^20^, Protein Data Bank (PDB)^21^, ChEMBL^19^, DrugBank^16^ or the Kyoto Encyclopedia of Genes and Genomes (KEGG)^22^. For the data *Bioassay results*, four types of bioassay data, i.e., the inhibitor constant (K_i_), half-maximal inhibitory concentration (IC_50_), half-maximal effective concentration (EC_50_) and dissociation constant (K_d_), were recorded according to the actual bioassay models. The final list of small molecules and targets, along with their collected chemical/biological properties, are shown in Supplementary Dataset 1.

#### Models

We manually consulted the bioassays and analysed the experimental methods, which were then categorised into 16 experimental models (Additional File 3). These experimental models, such as competitive binding assay, cytotoxicity assay, and enzyme activity assay, were appointed to each protein target and assigned with a unique model ID.

The data were stored in a MariaDB (5.5.52) database. The AICD was implemented as a PHP-based Internet application deployed to an Apache Tomcat server (PHP 5.4.16 and HTTPD 2.4.6). Thus, the AICD can be accessed freely *via* the Internet.

### Analyses of chemical space and comparison of molecular descriptors

Molecular descriptors can represent the properties of molecules. Principal component analysis (PCA) can reduce the dimensions of these descriptors. Then, a chemical space can be constructed to compare the similarity between small molecules in the AICD and US Food and Drug Administration (FDA)-approved drugs.

**Figure 1:**
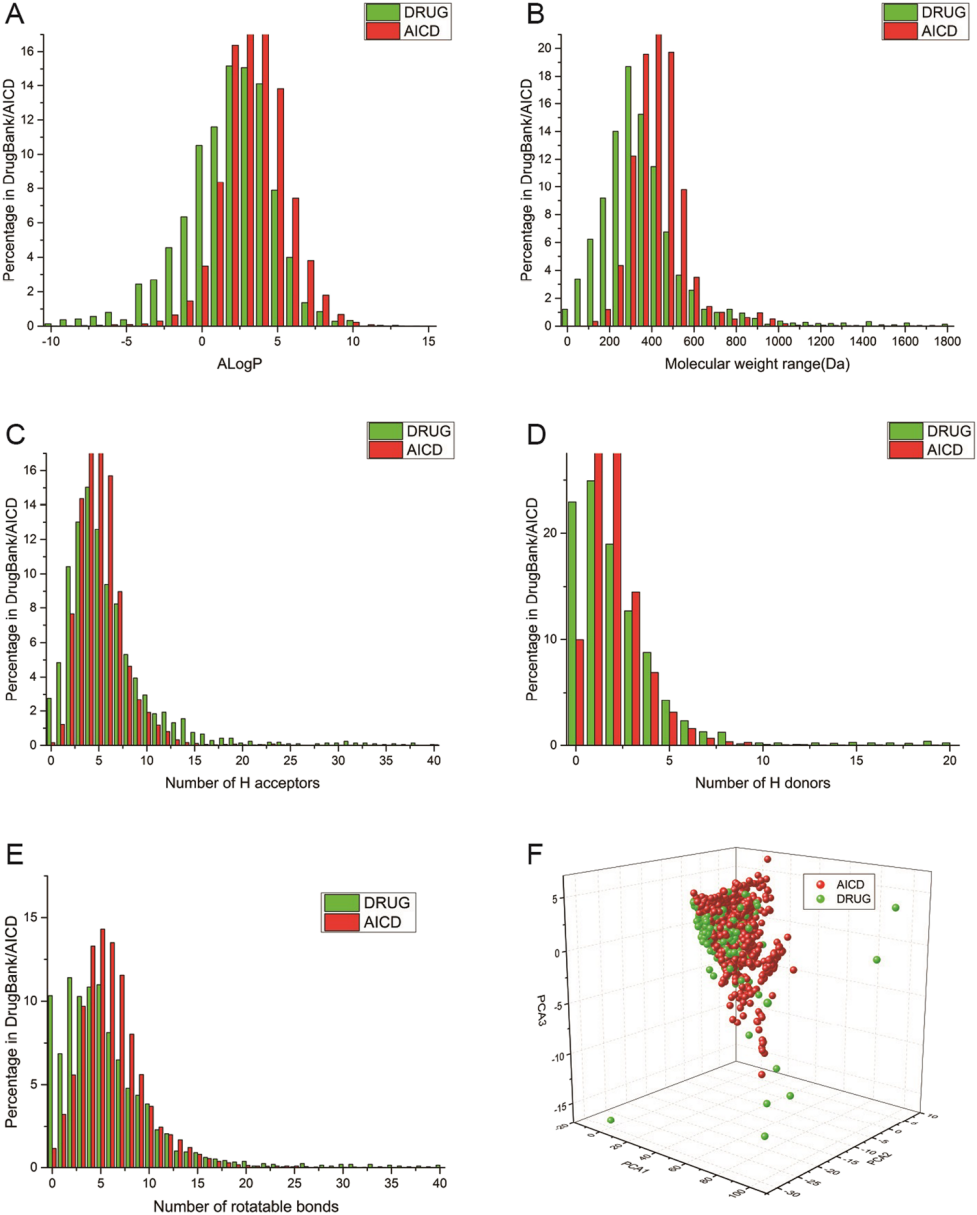
Analysis of chemical space. (**A**–**E**) Comparison of the distribution of five molecular descriptors between small molecules in the AICD and FDA-approved drugs. The chi-square test was undertaken: A (χ^2^ = 546, p = 0.255), B (χ^2^ = 651, p = 0.273), C (χ^2^ = 906.21, p = 3.90 × 10^−5^), D (χ^2^ = 281.75, p = 0.242), E (χ2 = 1035.6, p = 0.049). (**F**) Distribution of the chemical space of the small molecules in the AICD and FDA-approved drugs according to principal component analysis.

PCA was conducted in the Library Analysis module of Discovery Studio 2.5, which is a suite of software for simulating small-molecule and macromolecule systems (Accelrys, San Diego, CA, USA). PCA is an orthogonal linear transformation method used to convert data into a new coordinate system. Three principal components for each small molecule were obtained: PC1, PC2 and PC3. The variance of data maximised in the first coordinate is termed the “first principal component” (PC1). The remainder of the variance maximised in the second coordinate is termed the “second principal component” (PC2), and so on. The variance explained by PC1, PC2 and PC3 was 0.627, 0.188 and 0.083, respectively. PCA calculation results are displayed as a 3D scatter plot in Fig. 1F.

Five molecular descriptors of each molecule (the partition coefficient (ALogP), molecular weight, number of hydrogen bond acceptors, number of hydrogen bond donors, and the number of rotatable bonds) were applied for PCA. The structure file in sdf format of FDA-approved drugs was downloaded onto the DrugBank website (www.drugbank.ca/releases/latest#structures), and the molecular descriptors were calculated by Discovery Studio 2.5. The distribution of five molecular descriptors between molecules in the AICD and FDA-approved drugs were compared (Fig. 1A–E) and the chi-square test was carried out for each comparison.

### Analyses of molecular activity and small-molecule–target network of the AICD

**Figure 2:**
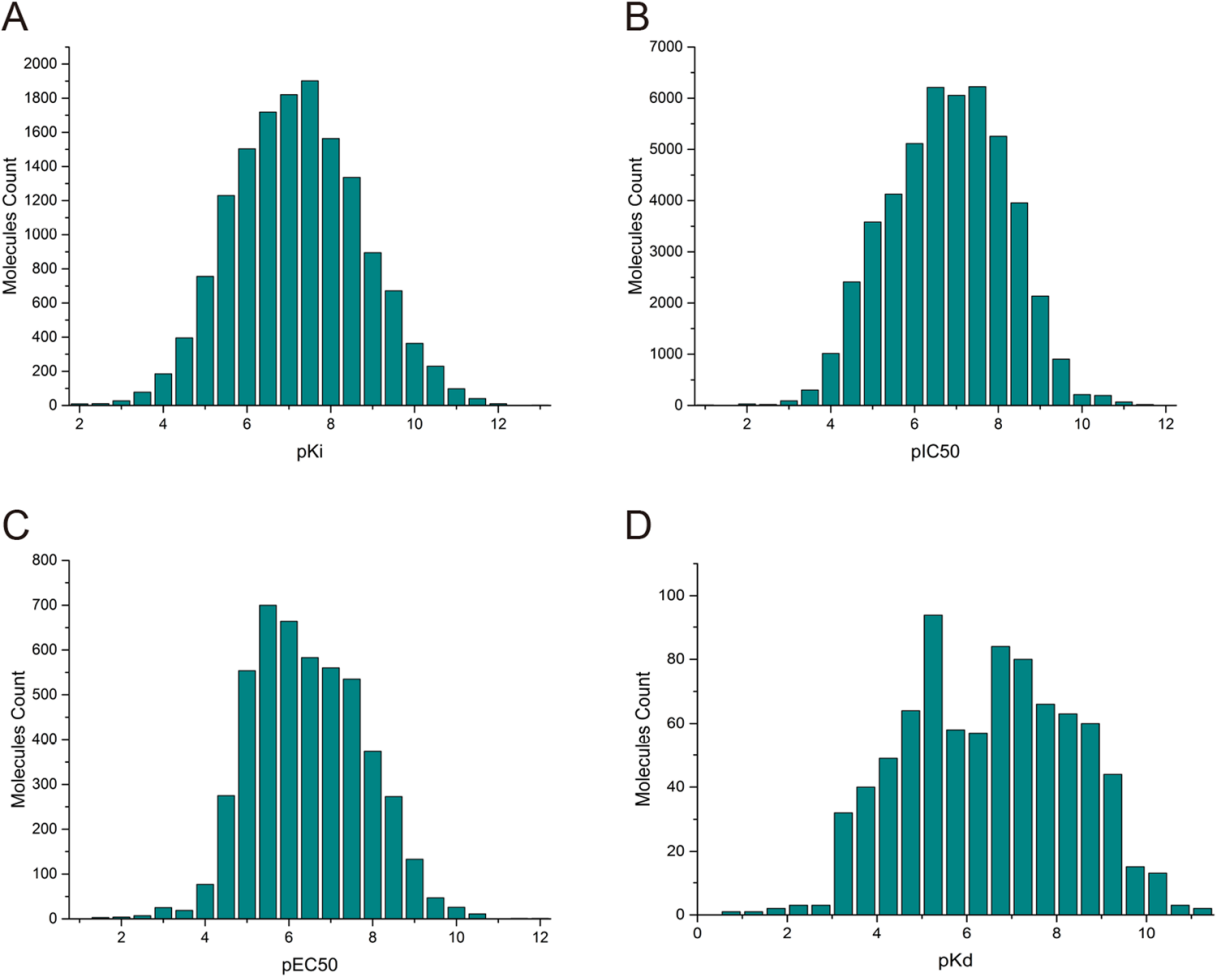
Distribution of bioassay results of AICD compounds.

Based on the library of small molecules in the AICD, the statistical distribution of bioassay results, such as K_i_, K_d_, IC_50_ and EC_50_, were analysed and plotted by Origin 9.1 (Fig. 2).

**Figure 3:**
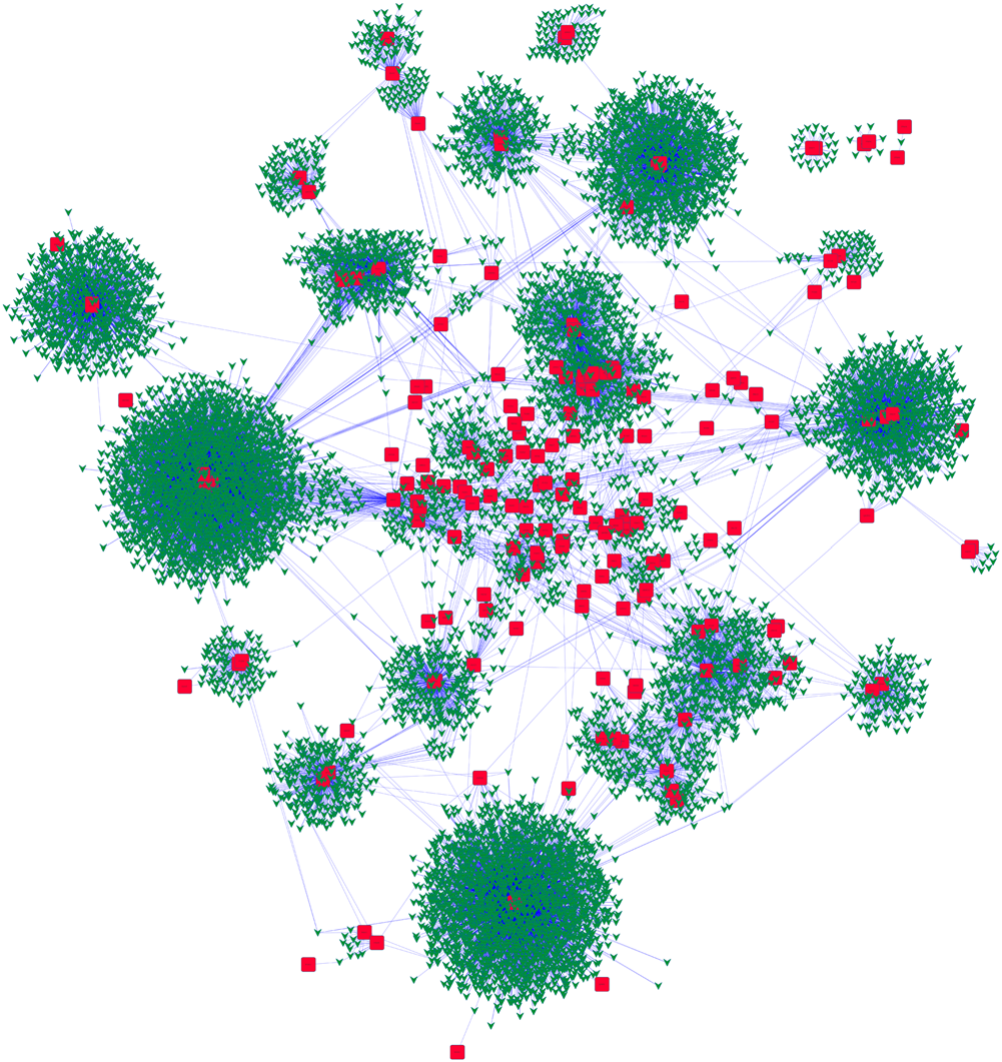
Small molecule–target network of the AICD. green triangles = molecules; red boxes = targets.

A small molecule–target interactions network was constructed and visualised by Cytoscape 3.2.1 (image not shown). The degree of each node was calculated by the Network Analyzer plugin, and the values of small molecules and targets of degree ≥2 were placed in Supplementary Dataset 2. To highlight the multi-target effects, small molecules with degree ≥2 were selected to construct the small molecule–target network (Fig. 3).

## Utility and Discussion

### Utility of the AICD

The AICD comprises 79781 small molecules and their identification information. The AICD can be accessed freely at http://956023.ichengyun.net/AICD/index.php. The AICD provides an intuitive interface. The Internet pages are divided into five sections: *Home*, *Browse*, *Search*, *Online Docking* and *About*. In the *Hom*e page, users can query the AICD *via* CAS Number, Molecule ID, Chemical Name, Molecular Formula, SMILES, InChiKey or Protein ID.

For instance, submitting a query for AICD00001 would generate a table of the structure, InChI Key, molecular formula, molecular weight, CAS Number, chemical name and download link. In the *Browse* section, users can browse through all the deposited molecules, targets and bioassay models in the AICD. In the *Search* section, the AICD can be queried by molecules, targets and models. Unlike the *Home* section, users can search for information using chemical properties in the *Search*/*molecule*, including *molecular weight*, *AlogP*, *number of hydrogen bond acceptors*, *number of hydrogen bond donors*, *number of rotatable bonds*, *number of rings* and *number of aromatic rings*. Meanwhile, the AICD also provides AutoDock 4.2.6 based on the Online Docking tool for computing the binding energy between a compound and an AICD target. In the Online Docking section, users can select a target and then upload the structure of a compound in mol2/sdf/pdb format or simply input an AICD compound ID. After job submission, the computing result is sent to users by email. Currently, our server receives a docking request of one compound and one target for each submission, but users can contact us for batch tasks. This strategy would greatly simplify the process of molecular docking for non-experts. In the *About* page, it is feasible to download the information of the AICD, including all anti-inflammatory compounds in a single sdf file, one anti-inflammatory compound in each sdf file, and a table of molecular information.

### Statistics of the molecular properties of small molecules and comparison between small molecules and FDA-approved drugs

The AICD database contains 79,781 molecules, and the number of FDA-approved drugs is 2,144. There is an overlap of 325 molecules between the small molecules in the AICD and FDA-approved drugs. They can be searched in the AICD by the DrugBank ID. The distribution of five molecular descriptors of AICD small molecules (*n* = 79,781) and FDA-approved drugs (*n* = 2,144) were compared (Fig. 1A–E). The histograms showed that the distributions of ALogP, molecular weight, hydrogen bond acceptors, hydrogen bond donors and rotatable bonds overlapped broadly between groups. With respect to molecular weight, the percentage of small molecules of size 0–300 Da was lower than that of FDA-approved drugs, but the percentage of small molecules of size 400–1000 Da was higher than that of FDA-approved drugs. With regard to ALogP, small molecules in the AICD had lower percentages than FDA-approved agents in the region −10 to + 2.5, but larger percentages in the region + 2.5 to + 10. The high percentages of ALogP obtained for small molecules suggested that small molecules would have high solubility. Solubility has considerable impact on therapeutic effectiveness, and the distribution of ALogP could provide useful information. The results of the chi-square test showed no significant difference in the properties of ALogP, molecular weight, or the number of hydrogen bond acceptors between AICD small molecules and FDA-approved drugs.

The concept of ‘chemical space’ is important for drug discovery^23–26^. ‘Chemical space’ refers to the space spanned by all possible molecules and chemical compounds adhering to a given set of construction principles and boundary conditions^27^. To obtain better understanding of small molecules in the AICD and FDA-approved agents, PCA was adopted to provide a visual illustration in chemical space. The three-dimensional plot in Fig. 1F provides an opportunity to compare the distribution between small molecules in the AICD and FDA-approved drugs. The wide distribution in chemical space indicates that there is a vast diversity for AICD small molecules. The overlap of chemical space with FDA-approved drugs suggests that small molecules in the AICD have good ‘druglikeness’, and that the AICD could be serviced as a large source for drug discovery. The statistics of 15 important molecular descriptors of small molecules in the AICD and FDA-approved drugs in DrugBank were calculated (Table 1). The mean ( ± standard deviation) values of 10 out of 15 molecular descriptors of small molecules were greater than those of FDA-approved drugs.

**Table 1:** Statistics of the molecular descriptors of molecules in the AICD and FDA-approved drugs in DrugBank.

Descriptor	Molecules in the AICD				Approved drugs			
	Mean	Median	Min	Max	Mean	Median	Min	Max
AlogP	3.36 ± 2.44	3.388	−27.50	26.68	1.665 ± 3.66	2.119	−69.0	20.1
MW	447.8 ± 246.2	421.8	68.1	5511.7	377.3 ± 364.6	320.8	4.0	7177
NRB	6.588 ± 5.96	6	0	166	6.3 ± 10.58	5	0	182
NR	3.88 ± 1.4	4	0	17	2.6 ± 2.2	2	0	46
NAR	2.690 ± 1.20	3	0	11	1.35 ± 1.25	1	0	8
NHA	5.372 ± 4.05	5	0	98	6.597 ± 9.6	5	0	191
NHD	2.230 ± 3.51	2	0	67	2.746 ± 5.7	2	0	116
MV	288.7 ± 150.1	271.0	42.5	3194.7	246.1 ± 228.2	218.1	2.7	4225.1
MSA	422.3 ± 242.3	395.5	75.98	5340.2	363.3 ± 349.4	313.0	12.6	6351
MPSA	101.5 ± 105.9	90.4	0	2578.6	104.1 ± 161.1	74.59	0	3227
MFPSA	0.236 ± 0.088	0.226	0	1.0	0.304 ± 0.22	0.25	0	1
MSASA	672.7 ± 307.0	638.6	224.9	6792.3	579.7 ± 431.5	524.3	138.0	7761
MPSASA	155.3 ± 165.2	136.0	0	3793.8	164.2 ± 243.8	124.1	0	4587
MFPSASA	0.225 ± 0.094	0.211	0	0.90	0.275 ± 0.183	0.239	0	0.895
MSAV	595.8 ± 267.4	566.7	198.1	6021.7	510.7 ± 376.0	462.9	124.4	7001.4

The descriptors of 79781 molecules in the AICD and 2144 FDA-approved small molecule drugs in DrugBank were calculated by Discovery Studio. MW: Molecular Weight; NRB: Number of Rotatable Bonds; NR: Number of Rings; NAR: Number of Aromatic Rings; NHA: Number of Hydrogen Bond Acceptors; NHD: Number of Hydrogen Bond Donors; MV: Molecular Volume; MSA: Molecular Surface Area; MPSA: Molecular Polar Surface Area; MFPSA: Molecular Fractional Polar Surface Area; MSASA: Molecular SASA; MPSASA: Molecular Polar SASA; MFPSASA: Molecular Fractional Polar SASA; MSAV: Molecular SAVol.

Lipinski’s “rule of five” (ro5) can be used to aid the discovery of good drug-like molecules from large libraries of compounds^28^. The ro5 describes four constraints for oral drugs: the molecular weight should be <500 Da; the number of hydrogen bond acceptors should be <10; the number of hydrogen bond donors should be <5; and ALogP should be <5. We found that 62.5% of all small molecules in the AICD satisfied all the conditions of the ro5. This result was comparable with that for FDA-approved drugs (68%) (Table 2).

**Table 2:** Statistics of satisfied conditions for Lipinski’s “rule of five” of molecules in the AICD and approved small drugs in DrugBank.

Rule of five	DrugBank (2144)	AICD_all (79781)
All satisfied	1457 (68.0%)	49888 (62.5%)
Except MW	1503 (70.1%)	56159 (70.4%)
Except hydrogen bond acceptors	1489 (69.4%)	50137 (62.8%)
Except hydrogen bond donors	1541 (71.9%)	51985 (65.2%)
Except AlogP	1556 (72.6%)	57562 (72.1%)

Based on the analysis of the molecular properties and the comparison with FDA-approved drugs, we found that AICD small molecules exhibited a high degree of similarity with FDA-approved drugs. This observation indicated that the small molecules in the AICD had high potential for drug development.

### Gene set enrichment analysis of AICD targets

**Figure 4:**
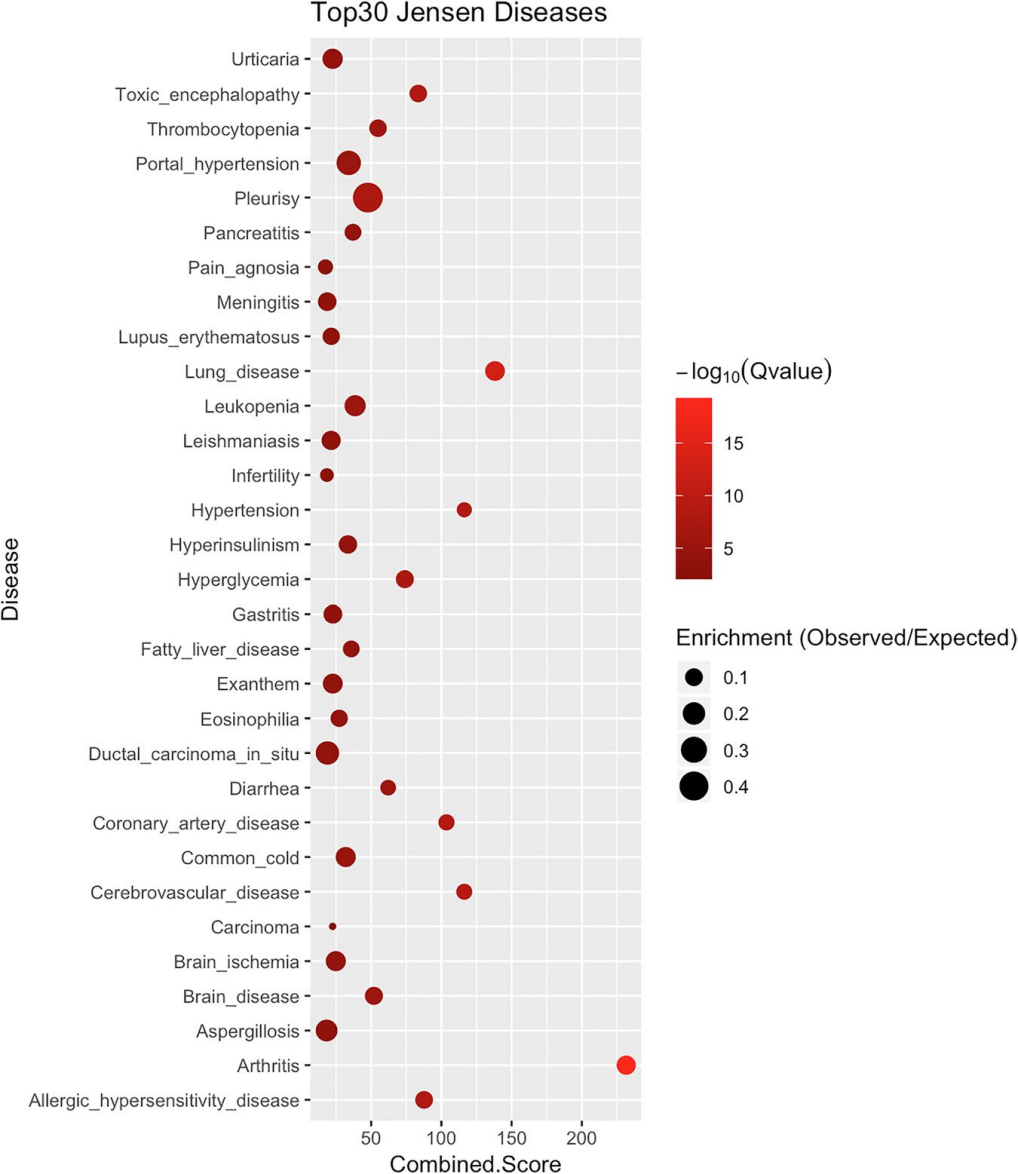
Disease–gene association analysis of AICD targets. Gene symbols of targets were uploaded for gene set enrichment analysis in Enrichr. The Top 30 diseases which might be associated with AICD targets were plotted.

Enrichment analysis was undertaken by the Enrichr online tool to explore which pathways, biological processes or diseases the targets we collected were related to. Enrichr integrates gene set enrichment analysis from multiple sources, including Reactome, Gene Ontology (GO) and DISEASES databases^29^. As expected, AICD targets were enriched for a series of inflammation-related pathways (Supplementary Dataset 3) or biological processes (Supplementary Dataset 4). The DISEASES database (http://diseases.jensenlab.org), which mines disease–gene associations from the literature, revealed that AICD targets were associated with various common human diseases (Fig. 4 and Supplementary Dataset 5). It was, therefore, expected that the compounds and their bioassay outcomes in the AICD might be applicable for translational studies aimed at treating various diseases.

### Activity analysis of the small molecules and small-molecule–target network of the AICD

In the AICD, we obtained 14858 records of K_i_, 47960 records of IC_50_, 4879 records of EC_50_, and 834 records of K_d_. The distributions of pK_i_, pIC_50_, pEC_50_ and pK_d_ are shown in Fig. 2. The highest abundance of small molecules for pK_i_ was between 6 and 8, and it was similar for pIC_50_. The proportion of small molecules with K_i_, IC_50_, EC_50_ and K_d_ values < 5000 nM was 89.4% (13276/14858), 83.4% (40002/47960), 78.9% (3849/4879), and 68.6% (572/834) respectively, suggesting that most of the small molecules in the AICD had good activities for inflammation-related targets. By analysing the activity of small molecules in the AICD database, we can obtain clues for the development of anti-inflammatory drugs.

By analysing all 79781 small molecules in the AICD using the Cytoscope plug-in network analyser, we obtained a list of the degree of interaction of small molecules and targets, and the network data are shown in Supplementary Dataset 6. The Cytoscape session file (.cys) containing this network is put in Supplementary Dataset 7. Small molecules of degree ≥2 have a poly-pharmacological effect, and can act on various anti-inflammatory targets. They may become multi-target drugs, and have a better therapeutic effect against inflammation than other drugs. In relative terms, they are core molecules in the AICD, so small molecules of degree ≥2 and their corresponding targets were chosen to form a core compound–target network (Fig. 3), and the network data are shown in Supplementary Dataset 8. The Cytoscape session file (.cys) containing this network is put in Supplementary Dataset 9. There are 11405 small molecules with degree ≥2, suggesting that 14.3% of small molecules in the AICD can interact with at least two inflammation-related targets. A total of 79 small molecules in the AICD have ≥10 targets. Notably, three compounds, i.e., AICD00516 (staurosporine), AICD00814 (gefitinib) and AICD00947 (erlotinib), have 16 targets. In the AICD, the recorded molecules targeted at an average of 1.19 protein targets, and each target protein contained an average of 455.9 hits. The evidence stated above suggests that many molecules in the AICD have multi-target effects, and that the AICD database can be a good resource for discovery of anti-inflammatory drugs.

### Case study of the AICD: pathway-based screening for drug combination/multi-target drug

A ‘multi-target drug’ is a molecule that acts on multiple proteins that are linked in pathways or biological processes, or are associated with a disease. A ‘drug combination’ is a combination of multiple molecules acting on several targets to synergise in a biological process or disease. These are two of the current trends in drug development^30,31^. In this section, we used the AICD to make a preliminary screening of possible multi-target drugs and drug combination to provide a case study of the utility of the AICD.

Mammalian target of rapamycin (mTOR) integrates the input from upstream pathways, including insulin growth factor (IGF)-1 and IGF-2, and amino acids^32^. mTOR also ‘senses’ the nutrients, oxygen, and energy levels of cells^33^. Thus, the mTOR pathway is a central regulator of mammalian metabolism and physiology, with important roles in the function of tissues (e.g., liver, muscle, white and brown adipose tissue, brain) and is dysregulated in human diseases such as diabetes mellitus, obesity, depression, and certain cancers^34,35^.

**Figure 5:**
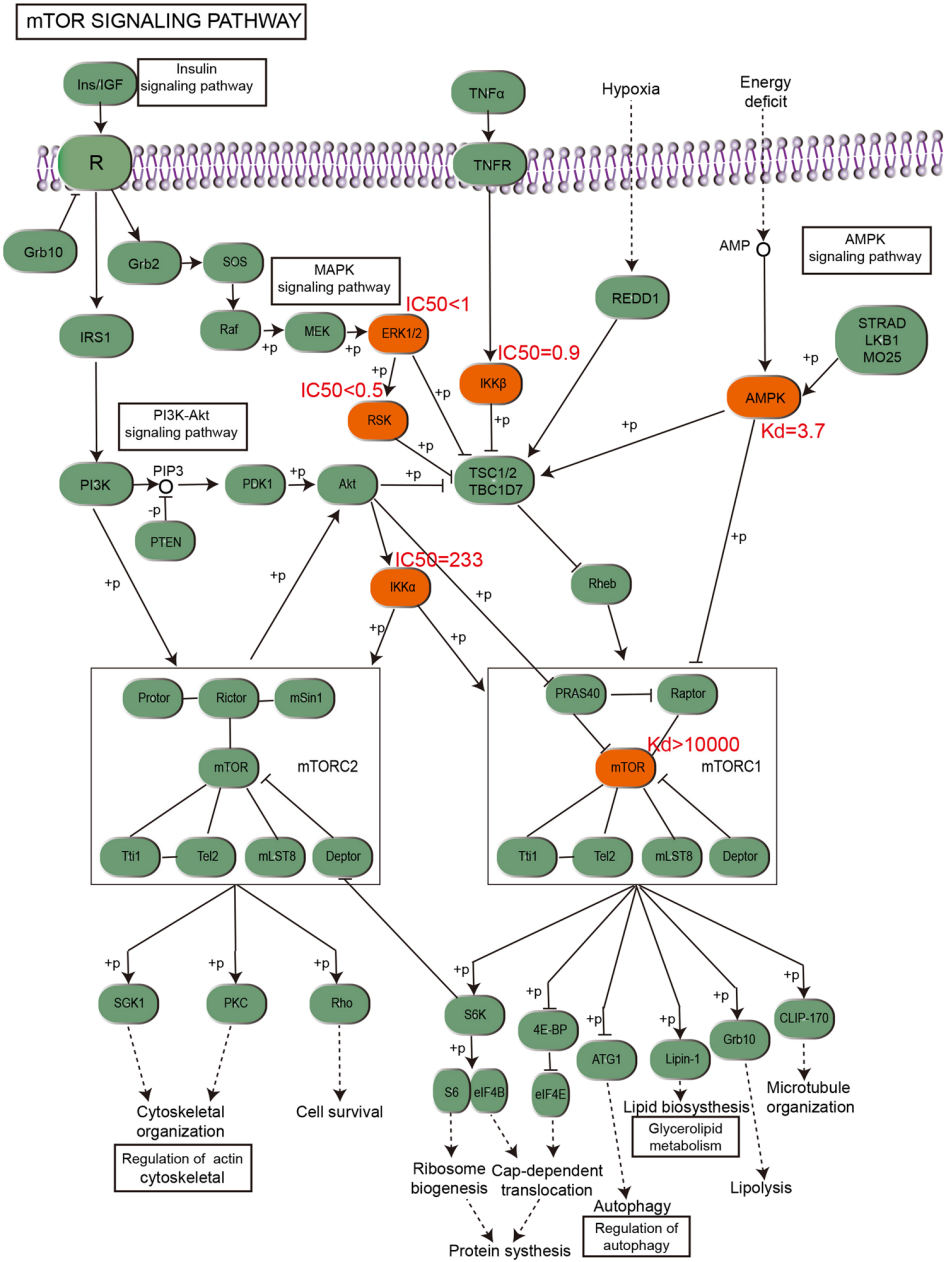
Human mTOR pathway. The orange boxes represent the targets that staurosporine (molecule ID: AICD00516) can act upon, and then we marked the relevant activity data next to these targets.

We used the mTOR pathway as an example to show how the AICD can be employed to promote the discovery of multi-target drugs and drug combinations. The degree values of molecules and targets in the AICD are in Supplementary Dataset 2, so molecules with several targets could be found readily. Then, the related targets could be searched directly on the AICD website or obtained from network data (Supplementary Dataset 6 or Supplementary Dataset 8). Then, we could obtain the relevant genes of the human mTOR pathway from the KEGG website, and then compared them with the target of the compounds in the AICD. Thus, it was found that the compound staurosporine (molecule ID: AICD00516) could act on multiple nodes in the mTOR pathway and had good biological activity. The orange boxes in Fig. 5 represent the nodes that staurosporine can interact with in the mTOR pathway. Hence, staurosporine would be a potential multi-target drug that could have a role in mTOR-related diseases.

Figure 5 also shows that 5′ adenosine monophosphate-activated protein kinase (AMPK) and mTOR are two important nodes in the pathway. Hence, if we want to develop a drug combination, we can start from these two targets. Then, we can discover their corresponding active small molecules readily from the AICD database through the Uniprot ID of these targets. Subsequently, we can select the small molecules with the best activity to download their structural files for further drug-like and ADME/T analysis(ADMET is a comprehensive study of the absorption, distribution, metabolism, excretion and toxicity of drugs), and then test their anti-inflammatory biological activity. Therefore, the AICD can promote the development of drug combinations for disease treatments.

## Conclusions

Inflammation is associated with various diseases. The AICD collected 79,781 small molecules which had been tested for anti-inflammatory activity on 232 targets. A total of 122,804 pieces of bioassay results were extracted from 1031 experimental records and assigned to 16 categories of bioassay models. The anti-inflammatory small molecules in the AICD had good drug-like properties and structural diversity, as indicated by the distribution of chemistry descriptors and chemical space. The targets in the AICD were closely related to the vast majority of inflammatory pathways and common human diseases by gene enrichment analysis.

Development of the AICD could promote study of the mechanism of action of anti-inflammatory drugs and the discovery of new drugs. Molecules that act on the same target could be used to: (i) construct a quantitative structure–activity relationship model of this target; (ii) predict the activity of unknown molecules. Drugs acting on different targets could be used for screening of drug combinations or exploration of pharmacological systems.

We also developed an online molecular docking system that allows users to carry out calculations on molecular docking for small molecules and target proteins in the AICD database to predict their affinity, which greatly simplifies the process of molecular docking for non-experts. The AICD reveals several features of small molecules with anti-inflammatory properties that could be used for drug discovery. By identifying the associations between small molecules and cellular target proteins, the AICD may aid the discovery of novel anti-inflammatory compounds.

## Supplementary information

supplementary information

Dataset 1

Dataset 2

Dataset 3

Dataset 4

Dataset 5

Dataset 6

Dataset 7

Dataset 8

Dataset 9

## Data Availability

The AICD can be accessed free at http://956023.ichengyun.net/AICD/index.php. The AICD website has been tested with latest versions of several browsers, including Opera, Internet Explorer Edge, Firefox and Google Chrome. All the data in the AICD were distributed under the terms and conditions of the Creative Commons Attribution (CC BY) license (http://creativecommons.org/licenses/by/4.0/).
