# Supplementary material for: AICD: an integrated anti-inflammatory compounds database for drug discovery
Source: Sci Rep. 2019 May 23;9:7737. doi: 10.1038/s41598-019-44227-x (PMC6533287; doi:10.1038/s41598-019-44227-x)
Supplement: Supplementary file 1 — supplementary information [file 41598_2019_44227_MOESM1_ESM.docx]

**AICD: an integrated** **anti-inflammatory compounds database for drug discovery**

Kun Wang^1,2†^, Jianyong Xiao^3†^, Xiaodong Liu^4†^, Zhuqiao Jiang^3^, Yujuan Zhan^3^, Ting Yin^3^, Lina He^2^, Fangyuan Zhang^2^, Shangping Xing^1^, Bonan Chen^3^, Yingshi Li^2^, Fengxue Zhang^1^, Zaoyuan Kuang^1*^, Biaoyan Du^2*^ and Jiangyong Gu^5*^

1 Research Center of Integrative Medicine, School of Basic Medical Science, Guangzhou University of Chinese Medicine, Guangzhou 510006, China; wk1299461695@126.com (K.W.); shopingxing@gmail.com (S.X.); zhangfengxue@gzucm.edu.cn (F.Z.)

2 Department of Pathology, Guangzhou University of Chinese Medicine, Guangzhou 510006, China;

3244415934@qq.com (L.H.); zh-p-y@163.com (F.Z.); 1102925623@qq.com (Y.L.)

3 Department of Biochemistry, Guangzhou University of Chinese Medicine, Guangzhou 510006, China; jianyongxiao@163.com (J.X); jiangzhuqiao597@163.com (Z.J); yujuan_zhan@163.com (Y.Z); 2313260212@qq.com (T.Y.); 15626145866@163.com (B.C.)

4 Department of Anaesthesia and Intensive Care, The Chinese University of Hong Kong, Hong Kong 999077, China; greg.xd.lau@gmail.com (X.L.)

5 The Second Clinical College, Guangzhou University of Chinese Medicine, Guangzhou 510006, China

* Correspondence:

gujy@gzucm.edu.cn (J.G.); dubiaoyan@gzucm.edu.cn (B.D.); zykuang@gzucm.edu.cn (Z.K.);

† These authors contributed equally to this work.

**Additional file 1 Inflammatory targets and the methods for targets collection**

These publications are mainly about QSAR (Quantitative structure–activity relationship). QSAR is based on molecules with known activity on a target to create a model to predict the activity of unknown molecules. According to the definition of QSAR, the articles with QSAR definitely have information on the targets and related molecules. We searched PubMed using QSAR and inflammation/inflammatory as keywords for the targets of interest. We noticed that the molecular information of many compounds is either missed or ambiguous in these publications. Therefore, these QSAR related literatures were reviewed mainly for targets extraction. For extracting molecular information, the methodology was described in the *Molecules and bioassay results* of the manuscript. To include the utmost number of targets, we have also review a series of non-QSAR related publications. This generated a total of 153 non-redundant targets which were listed in the table a. The PubMed IDs of these source literatures were also included in the table a.

Curator 1 collected the targets from the publications. Curator 2 determined whether the targets were related to inflammation. Then Dr. Liu made a final decision. The decisions by curators and Dr. Liu were recorded in the table b.

a.The final targets list and PubMed IDs of related publications

| Target Name | PMID |
| --- | --- |
| 3-phosphoinositide-dependent protein kinase 1 | 18717540 |
| 5'-AMP-activated protein kinase catalytic subunit alpha-1 | 24123051 |
| 5'-AMP-activated protein kinase catalytic subunit alpha-2 | 21673972 |
| 5'-AMP-activated protein kinase subunit beta-1 | 22080866 |
| 5'-AMP-activated protein kinase subunit beta-2 | 22080866 |
| 5'-AMP-activated protein kinase subunit gamma-1 | 17438370 |
| 5'-AMP-activated protein kinase subunit gamma-2 | 19438609 |
| 5'-AMP-activated protein kinase subunit gamma-3 | 24638063 |
| 78 kDa glucose-regulated protein | 19503733 |
| Acetyl-CoA acetyltransferase, mitochondrial | 17483192 |
| Acid-sensing ion channel 1 | 17994101 |
| Adenosine deaminase | 21138280 |
| Adenosine receptor A2a | 16012931 |
| Adenosine receptor A2b | 19012575 |
| Aldo-keto reductase family 1 member C1 | 27698389 |
| Aldose reductase | 23700991 |
| Aminopeptidase N | 12086480 |
| Androgen receptor | 21598194 |
| Annexin A1 | 19104500 |
| Apoptosis regulator Bcl-2 | 25935642 |
| Arachidonate 5-lipoxygenase | 22779798 |
| Arachidonate 5-lipoxygenase-activating protein | 18276139 |
| Arachidonate 5-lipoxygenase-activating protein | 20207999 |
| Aryl hydrocarbon receptor | 23424013 |
| Arylamine N-acetyltransferase | 12943195 |
| B1 bradykinin receptor | 22197141 |
| B1 bradykinin receptor | 10188975 |
| B2 bradykinin receptor | 10188975 |
| Bile salt export pump | 17181454 |
| cAMP-specific 3',5'-cyclic phosphodiesterase 4A | 11472206 |
| cAMP-specific 3',5'-cyclic phosphodiesterase 4B | 11472206 |
| cAMP-specific 3',5'-cyclic phosphodiesterase 4D | 11472206 |
| C-C chemokine receptor type 2 | 17364026 |
| C-C chemokine receptor type 3 | 9466968 |
| Cellular tumor antigen p53 | 30344124 |
| Chloride channel protein ClC-Ka | 20805576 |
| Complement C1q subcomponent subunit A | 22779798 |
| Complement C1q subcomponent subunit B | 18318990 |
| Complement C1q subcomponent subunit C | 18318990 |
| Complement C1r subcomponent | 23031212 |
| Complement C1s subcomponent | 23031212 |
| Corticosteroid 11-beta-dehydrogenase isozyme 1 | 17631244 |
| C-X-C chemokine receptor type 1 | 25935642 |
| C-X-C chemokine receptor type 2 | 17958344 |
| C-X-C chemokine receptor type 4 | 17958344 |
| Cysteinyl leukotriene receptor 1 | 27908761 |
| Cysteinyl leukotriene receptor 2 | 9871597 |
| Cystic fibrosis transmembrane conductance regulator | 28148462 |
| Cystine/glutamate transporter | 17897437 |
| Cytosolic phospholipase A2 | 23103940 |
| Dihydroorotate dehydrogenase , mitochondrial | 23714018 |
| Egl nine homolog 1 | 21530741 |
| Endoplasmin | 25901531 |
| Endothelin-1 receptor | 14718401 |
| Epidermal growth factor receptor | 29115879 |
| Estrogen receptor beta | 27692995 |
| Farnesyl pyrophosphate synthase | 23979193 |
| Fatty acid-binding protein, intestinal | 28148462 |
| fMet-Leu-Phe receptor | 11714831 |
| Glucocorticoid receptor | 20334371 |
| Glutathione S-transferase | 16162009 |
| Glutathione S-transferase A2 | 24707136 |
| Group IIE secretory phospholipase A2 | 25755141 |
| Heat shock protein HSP 90-alpha | 19183457 |
| Hematopoietic prostaglandin D synthase | 18077391 |
| High affinity immunoglobulin gamma Fc receptor I | 26497521 |
| High affinity nerve growth factor receptor | 10764711 |
| Histamine H1 receptor | 27643714 |
| Histamine H4 receptor | 24495018 |
| Histone deacetylase 1 | 21570914 |
| Inhibitor of nuclear factor kappa-B kinase subunit alpha | 10807044 |
| Inhibitor of nuclear factor kappa-B kinase subunit beta | 14579522 |
| Inosine-5'-monophosphate dehydrogenase 1 | 12381354 |
| Inosine-5'-monophosphate dehydrogenase 2 | 21311411 |
| Integrin alpha-L | 17906117 |
| Interferon gamma | 25302700 |
| Interleukin-1 receptor-associated kinase 1 | 16024789 |
| Interleukin-1 receptor-associated kinase 4 | 26403930 |
| Interleukin-2 | 26051521 |
| Interleukin-8 ligand 8) | 15801837 |
| Kit ligand | 16483568 |
| Lactotransferrin | 22136726 |
| Lactoylglutathione lyase | 21237663 |
| Leukotriene A-4 hydrolase hydrolase) | 23220644 |
| Low affinity immunoglobulin gamma Fc region receptor II-a | 26089602 |
| Low affinity immunoglobulin gamma Fc region receptor II-b | 11525942 |
| Low affinity immunoglobulin gamma Fc region receptor II-c | 26089602 |
| Low affinity immunoglobulin gamma Fc region receptor III-A | 23680410 |
| Low affinity immunoglobulin gamma Fc region receptor III-B | 25778799 |
| Lymphotoxin-alpha | 29541795 |
| Melanocortin receptor 4 | 18625277 |
| Mineralocorticoid receptor | 24656565 |
| Mitogen-activated protein kinase 1 | 25064438 |
| Mitogen-activated protein kinase 3 | 28274926 |
| Mitogen-activated protein kinase 8 | 21276204 |
| Myeloperoxidase | 20199096 |
| NF-kappa-B inhibitor alpha | 11134171 |
| Nicotinamide phosphoribosyltransferase | 28610984 |
| Nitric oxide synthase, brain | 19125620 |
| Nitric oxide synthase, endothelial | 12086480 |
| Nitric oxide synthase, inducible | 27272764 |
| Nuclear factor NF-kappa-B p100 subunit | 22659375 |
| Nuclear factor NF-kappa-B p105 subunit | 10207726 |
| P2X purinoceptor 3 | 11069182 |
| Peptidyl-prolyl cis-trans isomerase FKBP1A | 25445495 |
| Peroxisome proliferator-activated receptor alpha | 19243388 |
| Peroxisome proliferator-activated receptor delta | 17512197 |
| Peroxisome proliferator-activated receptor gamma | 12071662 |
| Phospholipase A2 | 16967904 |
| Phospholipase A2, membrane associated | 23979193 |
| Potassium voltage-gated channel subfamily KQT member 2 | 16321582 |
| Potassium voltage-gated channel subfamily KQT member 3 | 23658954 |
| Progesterone receptor | 15932748 |
| Prokineticin receptor 2 | 17324478 |
| Prostacyclin synthase | 21081215 |
| Prostaglandin D2 receptor | 25437505 |
| Prostaglandin D2 receptor 2 | 21508345 |
| Prostaglandin E2 receptor EP4 subtype | 16966471 |
| Prostaglandin G/H synthase 1 | 8521478 |
| Prostaglandin G/H synthase 2 | 8140262 |
| Prostaglandin reductase 2 | 21508345 |
| Protein S100-A1 | 15149869 |
| Protein S100-A12 | 12832707 |
| Protein S100-A13 | 15838637 |
| Protein S100-A2 | 15149869 |
| Protein S100-B | 11578775 |
| Proteinase-activated receptor 2 | 10655102 |
| Protein-tyrosine kinase 2-beta | 16581827 |
| Retinoic acid receptor alpha | 12071662 |
| Retinoic acid receptor beta | 12071662 |
| Retinoic acid receptor gamma | 15615532 |
| Retinoic acid receptor RXR-alpha | 12071662 |
| Retinoic acid receptor RXR-beta | 12071662 |
| Retinoic acid receptor RXR-gamma | 12071662 |
| Ribosomal protein S6 kinase alpha-3 | 17481552 |
| Serine/threonine-protein kinase mTOR | 19362054 |
| Smoothened homolog | 29807798 |
| Sodium channel protein type 4 subunit alpha | 16919992 |
| Sphingosine kinase 1 | 28121178 |
| Squalene monooxygenase | 19811923 |
| Thiopurine S-methyltransferase | 20593505 |
| Thrombomodulin | 27925234 |
| Thromboxane A2 receptor | 20666724 |
| Thromboxane-A synthase | 19766029 |
| Tissue-type plasminogen activator | 18396699 |
| Toll-like receptor 4 | 28108356 |
| Toll-like receptor 9 | 25901531 |
| Transient receptor potential cation channel subfamily V member 1 | 27255083 |
| Tumor necrosis factor | 25003344 |
| Tumor necrosis factor receptor superfamily member 1B | 11904678 |
| Tyrosine-protein kinase JAK3 | 23368101 |
| UDP-glucuronosyltransferase 1-9 | 17200831 |
| Uncharacterized oxidoreductase CzcO-like | 22838648 |

b. The records of two curators and final judgements by Dr. Liu

| Target Name | Curator 1 | | Curator 2 | | Dr. Liu |
| --- | --- | --- | --- | --- | --- |
| Cysteinyl leukotriene receptor 2 | ZQJ | √ | LNH | √ | √ |
| cAMP-specific 3',5'-cyclic phosphodiesterase 4D | ZQJ | √ | LNH | √ | √ |
| cAMP-specific 3',5'-cyclic phosphodiesterase 4A | ZQJ | √ | LNH | √ | √ |
| cAMP-specific 3',5'-cyclic phosphodiesterase 4B | ZQJ | √ | LNH | √ | √ |
| Retinoic acid receptor beta | ZQJ | √ | LNH | √ | √ |
| Beta-1,4-galactosyltransferase 5 | ZQJ | √ | LNH | × | × |
| Retinoic acid receptor RXR-gamma | ZQJ | √ | LNH | √ | √ |
| Retinoic acid receptor RXR-beta | ZQJ | √ | LNH | √ | √ |
| Retinoic acid receptor alpha | ZQJ | √ | LNH | √ | √ |
| Retinoic acid receptor RXR-alpha | ZQJ | √ | LNH | √ | √ |
| Peroxisome proliferator-activated receptor gamma | ZQJ | √ | LNH | √ | √ |
| Nitric oxide synthase, endothelial | ZQJ | √ | LNH | √ | √ |
| Aminopeptidase N | ZQJ | √ | LNH | √ | √ |
| Arylamine N-acetyltransferase | ZQJ | √ | LNH | √ | √ |
| Retinoic acid receptor gamma | ZQJ | √ | LNH | √ | √ |
| Interleukin-8 ligand 8) | ZQJ | √ | LNH | √ | √ |
| Glutathione S-transferase | ZQJ | √ | LNH | √ | √ |
| HLA-DR4 protein | ZQJ | √ | LNH | × | × |
| Phospholipase A2 | ZQJ | √ | LNH | √ | √ |
| Bile salt export pump | ZQJ | √ | LNH | √ | √ |
| Prokineticin receptor 2 | ZQJ | √ | LNH | √ | √ |
| Peroxisome proliferator-activated receptor delta | ZQJ | √ | LNH | √ | √ |
| C-X-C chemokine receptor type 4 | ZQJ | √ | LNH | √ | √ |
| C-X-C chemokine receptor type 2 | ZQJ | √ | LNH | √ | √ |
| Matrix metalloproteinase-16 | ZQJ | √ | LNH | × | × |
| Arachidonate 5-lipoxygenase-activating protein | ZQJ | √ | LNH | √ | √ |
| Tissue-type plasminogen activator | ZQJ | √ | LNH | √ | √ |
| 3-phosphoinositide-dependent protein kinase 1 | ZQJ | √ | LNH | √ | √ |
| Adenosine receptor A2b | ZQJ | √ | LNH | √ | √ |
| Nitric oxide synthase, brain | ZQJ | √ | LNH | √ | √ |
| Peroxisome proliferator-activated receptor alpha | ZQJ | √ | LNH | √ | √ |
| Squalene monooxygenase | ZQJ | √ | LNH | √ | √ |
| Myeloperoxidase | ZQJ | √ | LNH | √ | √ |
| Glucocorticoid receptor | ZQJ | √ | LNH | √ | √ |
| Thromboxane A2 receptor | ZQJ | √ | LNH | √ | √ |
| Receptor-interacting serine/threonine-protein kinase 2 | ZQJ | √ | LNH | × | × |
| Chloride channel protein ClC-Ka | ZQJ | √ | LNH | √ | √ |
| Prostacyclin synthase | ZQJ | √ | LNH | √ | √ |
| Adenosine deaminase | ZQJ | √ | LNH | √ | √ |
| Lactoylglutathione lyase | ZQJ | √ | LNH | √ | √ |
| Mitogen-activated protein kinase 8 | ZQJ | √ | LNH | √ | √ |
| Androgen receptor | ZQJ | √ | LNH | √ | √ |
| B1 bradykinin receptor | ZQJ | √ | LNH | √ | √ |
| Nuclear factor NF-kappa-B p100 subunit | ZQJ | √ | LNH | √ | √ |
| Complement C1q subcomponent subunit A | ZQJ | √ | LNH | √ | √ |
| Arachidonate 5-lipoxygenase | ZQJ | √ | LNH | √ | √ |
| Uncharacterized oxidoreductase CzcO-like | ZQJ | √ | LNH | √ | √ |
| N-acylethanolamine-hydrolyzing acid amidase | ZQJ | √ | LNH | × | × |
| Cytosolic phospholipase A2 | ZQJ | √ | LNH | √ | √ |
| Advanced glycosylation end product-specific receptor | ZQJ | √ | LNH | × | × |
| Leukotriene A-4 hydrolase hydrolase) | ZQJ | √ | LNH | √ | √ |
| Tyrosine-protein kinase JAK3 | ZQJ | √ | LNH | √ | √ |
| Aryl hydrocarbon receptor | ZQJ | √ | LNH | √ | √ |
| Potassium voltage-gated channel subfamily KQT member 3 | ZQJ | √ | LNH | √ | √ |
| Aldose reductase | ZQJ | √ | LNH | √ | √ |
| Dihydroorotate dehydrogenase , mitochondrial | ZQJ | √ | LNH | √ | √ |
| Solute carrier family 15 member 1 /peptide cotransporter) | ZQJ | √ | LNH | × | × |
| Farnesyl pyrophosphate synthase | ZQJ | √ | LNH | √ | √ |
| Phospholipase A2, membrane associated | ZQJ | √ | LNH | √ | √ |
| 5'-AMP-activated protein kinase catalytic subunit alpha-1 | ZQJ | √ | LNH | √ | √ |
| Histamine H4 receptor | ZQJ | √ | LNH | √ | √ |
| Mineralocorticoid receptor | ZQJ | √ | LNH | √ | √ |
| Tumor necrosis factor | ZQJ | √ | LNH | √ | √ |
| Mitogen-activated protein kinase 1 | ZQJ | √ | LNH | √ | √ |
| Interferon gamma | ZQJ | √ | LNH | √ | √ |
| Prostaglandin D2 receptor | ZQJ | √ | LNH | √ | √ |
| Group IIE secretory phospholipase A2 | ZQJ | √ | LNH | √ | √ |
| Low affinity immunoglobulin gamma Fc region receptor III-B | ZQJ | √ | LNH | √ | √ |
| Leukotriene C4 synthase synthase) | ZQJ | √ | LNH | × | × |
| Toll-like receptor 9 | ZQJ | √ | LNH | √ | √ |
| Endoplasmin | ZQJ | √ | LNH | √ | √ |
| C-X-C chemokine receptor type 1 | ZQJ | √ | LNH | √ | √ |
| Apoptosis regulator Bcl-2 | YJZ | √ | ZQJ | √ | √ |
| Interleukin-2 | YJZ | √ | ZQJ | √ | √ |
| Low affinity immunoglobulin gamma Fc region receptor II-a | YJZ | √ | ZQJ | √ | √ |
| Low affinity immunoglobulin gamma Fc region receptor II-c | YJZ | √ | ZQJ | √ | √ |
| Interleukin-1 receptor-associated kinase 4 | YJZ | √ | ZQJ | √ | √ |
| Transient receptor potential cation channel subfamily V member 1 | YJZ | √ | ZQJ | √ | √ |
| Nitric oxide synthase, inducible | YJZ | √ | ZQJ | × | √ |
| Histamine H1 receptor | YJZ | √ | ZQJ | √ | √ |
| Estrogen receptor beta | YJZ | √ | ZQJ | √ | √ |
| Cysteinyl leukotriene receptor 1 | YJZ | √ | ZQJ | √ | √ |
| Thrombomodulin | YJZ | √ | ZQJ | √ | √ |
| Tyrosine-protein kinase ITK/TSK | YJZ | √ | ZQJ | × | × |
| Toll-like receptor 4 | YJZ | √ | ZQJ | √ | √ |
| Sphingosine kinase 1 | YJZ | √ | ZQJ | √ | √ |
| Fatty acid-binding protein, intestinal | YJZ | √ | ZQJ | √ | √ |
| Cystic fibrosis transmembrane conductance regulator | YJZ | √ | ZQJ | √ | √ |
| Mitogen-activated protein kinase 3 | YJZ | √ | ZQJ | √ | √ |
| Nicotinamide phosphoribosyltransferase | YJZ | √ | ZQJ | √ | √ |
| Epidermal growth factor receptor | YJZ | √ | ZQJ | √ | √ |
| Tumor necrosis factor receptor superfamily member 1B | YJZ | √ | ZQJ | √ | √ |
| High affinity immunoglobulin gamma Fc receptor I | YJZ | √ | ZQJ | √ | √ |
| Low affinity immunoglobulin gamma Fc region receptor III-A | YJZ | √ | ZQJ | √ | √ |
| Low affinity immunoglobulin gamma Fc region receptor II-b | YJZ | √ | ZQJ | √ | √ |
| Lymphotoxin-alpha | YJZ | √ | ZQJ | √ | √ |
| Complement C1s subcomponent | YJZ | √ | ZQJ | √ | √ |
| Complement C1r subcomponent | YJZ | √ | ZQJ | √ | √ |
| Complement C1q subcomponent subunit B | YJZ | √ | ZQJ | √ | √ |
| Complement C1q subcomponent subunit C | YJZ | √ | ZQJ | √ | √ |
| Prostaglandin G/H synthase 2 | YJZ | √ | ZQJ | √ | √ |
| Prostaglandin G/H synthase 1 | YJZ | √ | ZQJ | √ | √ |
| Inhibitor of nuclear factor kappa-B kinase subunit alpha | YJZ | √ | ZQJ | √ | √ |
| Inhibitor of nuclear factor kappa-B kinase subunit beta | YJZ | √ | ZQJ | √ | √ |
| Prostaglandin reductase 2 | YJZ | √ | ZQJ | √ | √ |
| Prostaglandin D2 receptor 2 | YJZ | √ | ZQJ | √ | √ |
| Serine/threonine-protein kinase mTOR | YJZ | √ | ZQJ | √ | √ |
| Peptidyl-prolyl cis-trans isomerase FKBP1A | YJZ | √ | ZQJ | × | √ |
| Sodium channel protein type 4 subunit alpha | YJZ | √ | ZQJ | √ | √ |
| Acid-sensing ion channel 1 | YJZ | √ | ZQJ | √ | √ |
| Potassium voltage-gated channel subfamily KQT member 2 | YJZ | √ | ZQJ | √ | √ |
| Progesterone receptor | YJZ | √ | ZQJ | √ | √ |
| Glutathione S-transferase A2 | YJZ | √ | ZQJ | √ | √ |
| Corticosteroid 11-beta-dehydrogenase isozyme 1 | YJZ | √ | ZQJ | √ | √ |
| pH-sensing regulatory factor of peptide transporter | YJZ | √ | ZQJ | × | × |
| Inosine-5'-monophosphate dehydrogenase 1 | YJZ | √ | ZQJ | √ | √ |
| Inosine-5'-monophosphate dehydrogenase 2 | YJZ | √ | ZQJ | √ | √ |
| fMet-Leu-Phe receptor | YJZ | √ | ZQJ | √ | √ |
| Heat shock protein HSP 90-alpha | YJZ | √ | ZQJ | √ | √ |
| Annexin A1 | LNH | √ | YJZ | √ | √ |
| Protein S100-A1 | LNH | √ | YJZ | √ | √ |
| Protein S100-A12 | LNH | √ | YJZ | √ | √ |
| Protein S100-B | LNH | √ | YJZ | √ | √ |
| Protein S100-A13 | LNH | √ | YJZ | √ | √ |
| Protein S100-A2 | LNH | √ | YJZ | √ | √ |
| Acetyl-CoA acetyltransferase, mitochondrial | LNH | √ | YJZ | √ | √ |
| Thromboxane-A synthase | LNH | √ | YJZ | √ | √ |
| Aldo-keto reductase family 1 member C1 | LNH | √ | YJZ | × | √ |
| 5'-AMP-activated protein kinase catalytic subunit alpha-2 | LNH | √ | YJZ | √ | √ |
| 5'-AMP-activated protein kinase subunit beta-1 | LNH | √ | YJZ | √ | √ |
| 5'-AMP-activated protein kinase subunit beta-2 | LNH | √ | YJZ | √ | √ |
| 5'-AMP-activated protein kinase subunit gamma-1 | LNH | √ | YJZ | √ | √ |
| 5'-AMP-activated protein kinase subunit gamma-2 | LNH | √ | YJZ | √ | √ |
| 5'-AMP-activated protein kinase subunit gamma-3 | LNH | √ | YJZ | √ | √ |
| Endothelin-1 receptor | LNH | √ | YJZ | √ | √ |
| Cellular tumor antigen p53 | LNH | √ | YJZ | √ | √ |
| 78 kDa glucose-regulated protein | LNH | √ | YJZ | √ | √ |
| Ribosomal protein S6 kinase alpha-3 | LNH | √ | YJZ | √ | √ |
| NF-kappa-B inhibitor alpha | LNH | √ | YJZ | √ | √ |
| Nuclear factor NF-kappa-B p105 subunit | LNH | √ | YJZ | √ | √ |
| Smoothened homolog | LNH | √ | YJZ | √ | √ |
| Protein-tyrosine kinase 2-beta | LNH | √ | YJZ | √ | √ |
| Thiopurine S-methyltransferase | LNH | √ | YJZ | √ | √ |
| UDP-glucuronosyltransferase 1-9 | LNH | √ | YJZ | √ | √ |
| Lactotransferrin | LNH | √ | YJZ | √ | √ |
| B2 bradykinin receptor | LNH | √ | YJZ | √ | √ |
| Hematopoietic prostaglandin D synthase | LNH | √ | YJZ | √ | √ |
| Calcium/calmodulin-dependent protein kinase type II subunit delta | LNH | √ | YJZ | × | × |
| Prostaglandin E2 receptor EP4 subtype | LNH | √ | YJZ | √ | √ |
| Kit ligand | LNH | √ | YJZ | √ | √ |
| Proteinase-activated receptor 2 | LNH | √ | YJZ | √ | √ |
| B1 bradykinin receptor | LNH | √ | YJZ | √ | √ |
| Melanocortin receptor 4 | LNH | √ | YJZ | √ | √ |
| Interleukin-1 receptor-associated kinase 1 | LNH | √ | YJZ | √ | √ |
| Adenosine receptor A2a | LNH | √ | YJZ | √ | √ |
| C-C chemokine receptor type 2 | LNH | √ | YJZ | √ | √ |
| Egl nine homolog 1 | LNH | √ | YJZ | √ | √ |
| Arachidonate 5-lipoxygenase-activating protein | LNH | √ | YJZ | √ | √ |
| Integrin alpha-L | LNH | √ | YJZ | √ | √ |
| Solute carrier family 12 member 2 -chloride cotransporter 1) | LNH | √ | YJZ | × | × |
| C-C chemokine receptor type 3 | LNH | √ | YJZ | √ | √ |
| Histone deacetylase 1 | LNH | √ | YJZ | √ | √ |
| High affinity nerve growth factor receptor | LNH | √ | YJZ | √ | √ |
| P2X purinoceptor 3 | LNH | √ | YJZ | √ | √ |
| Cystine/glutamate transporter | LNH | √ | YJZ | √ | √ |

**Additional file 2 Anti-inflammatory drugs and their targets from DrugBank and TTD database**

| Target name | Uniprot ID | Drug name |
| --- | --- | --- |
| 3-phosphoinositide-dependent protein kinase 1 | O15530 | Celecoxib |
| 5'-AMP-activated protein kinase catalytic subunit alpha-1 | Q13131 | Acetylsalicylic acid |
| 5'-AMP-activated protein kinase catalytic subunit alpha-2 | P54646 | Acetylsalicylic acid |
| 5'-AMP-activated protein kinase subunit beta-1 | Q9Y478 | Acetylsalicylic acid |
| 5'-AMP-activated protein kinase subunit beta-2 | O43741 | Acetylsalicylic acid |
| 5'-AMP-activated protein kinase subunit gamma-1 | P54619 | Acetylsalicylic acid |
| 5'-AMP-activated protein kinase subunit gamma-2 | Q9UGJ0 | Acetylsalicylic acid |
| 5'-AMP-activated protein kinase subunit gamma-3 | Q9UGI9 | Acetylsalicylic acid |
| 78 kDa glucose-regulated protein | P11021 | Acetylsalicylic acid |
| Acetyl-CoA acetyltransferase, mitochondrial | P24752 | Sulfasalazine |
| Acid-sensing ion channel 1 | P78348 | Diclofenac |
| Aldo-keto reductase family 1 member C1 | Q04828 | Salicylic acid |
| Aldo-keto reductase family 1 member C1 | Q04828 | Acetylsalicylic acid |
| Aldose reductase | P15121 | Sulindac |
| Aminopeptidase N | P15144 | Icatibant |
| Androgen receptor | P10275 | Fludrocortisone |
| Annexin A1 | P04083 | Hydrocortisone |
| Annexin A1 | P04083 | Dexamethasone |
| Apoptosis regulator Bcl-2 | P10415 | Ibuprofen |
| Arachidonate 5-lipoxygenase | P09917 | Masoprocol |
| Arachidonate 5-lipoxygenase | P09917 | Mesalazine |
| Arachidonate 5-lipoxygenase | P09917 | Diclofenac |
| Arachidonate 5-lipoxygenase | P09917 | Zileuton |
| Arachidonate 5-lipoxygenase | P09917 | Sulfasalazine |
| Arachidonate 5-lipoxygenase | P09917 | Meclofenamic acid |
| Arachidonate 5-lipoxygenase | P09917 | Balsalazide |
| Aryl hydrocarbon receptor | P35869 | Leflunomide |
| Arylamine N-acetyltransferase | P0A5L8 | Mesalazine |
| B2 bradykinin receptor | P30411 | Icatibant |
| Bile salt export pump | O95342 | Clofazimine |
| cAMP-specific 3',5'-cyclic phosphodiesterase 4A | P27815 | Apremilast |
| cAMP-specific 3',5'-cyclic phosphodiesterase 4B | Q07343 | Apremilast |
| cAMP-specific 3',5'-cyclic phosphodiesterase 4D | Q08499 | Apremilast |
| Cellular tumor antigen p53 | P04637 | Acetylsalicylic acid |
| Chloride channel protein ClC-Ka | P51800 | Niflumic Acid |
| Complement C1q subcomponent subunit A | P02745 | Etanercept |
| Complement C1q subcomponent subunit A | P02745 | Adalimumab |
| Complement C1q subcomponent subunit B | P02746 | Etanercept |
| Complement C1q subcomponent subunit B | P02746 | Adalimumab |
| Complement C1q subcomponent subunit C | P02747 | Etanercept |
| Complement C1q subcomponent subunit C | P02747 | Adalimumab |
| Complement C1r subcomponent | P00736 | Etanercept |
| Complement C1r subcomponent | P00736 | Adalimumab |
| Complement C1s subcomponent | P09871 | Etanercept |
| Complement C1s subcomponent | P09871 | Adalimumab |
| Corticosteroid 11-beta-dehydrogenase isozyme 1 | P28845 | Prednisone |
| C-X-C chemokine receptor type 1 | P25024 | Ketoprofen |
| Cysteinyl leukotriene receptor 1 | Q9Y271 | Nedocromil |
| Cysteinyl leukotriene receptor 2 | Q9NS75 | Nedocromil |
| Cystic fibrosis transmembrane conductance regulator | P13569 | Ibuprofen |
| Cystine/glutamate transporter | Q9UPY5 | Sulfasalazine |
| Cytosolic phospholipase A2 | P47712 | Fluticasone Propionate |
| Cytosolic phospholipase A2 | P47712 | Niflumic Acid |
| Dihydroorotate dehydrogenase (quinone), mitochondrial | Q02127 | Leflunomide |
| Dihydroorotate dehydrogenase (quinone), mitochondrial | Q02127 | Teriflunomide |
| Endothelin-1 receptor | P25101 | Acetylsalicylic acid |
| Farnesyl pyrophosphate synthase | P14324 | Pamidronate |
| Fatty acid-binding protein, intestinal | P12104 | Ibuprofen |
| fMet-Leu-Phe receptor | P21462 | Nedocromil |
| Glucocorticoid receptor | P04150 | Flunisolide |
| Glucocorticoid receptor | P04150 | Diflorasone |
| Glucocorticoid receptor | P04150 | Alclometasone |
| Glucocorticoid receptor | P04150 | Medrysone |
| Glucocorticoid receptor | P04150 | Fluorometholone |
| Glucocorticoid receptor | P04150 | Beclomethasone dipropionate |
| Glucocorticoid receptor | P04150 | Betamethasone |
| Glucocorticoid receptor | P04150 | Desoximetasone |
| Glucocorticoid receptor | P04150 | Fluticasone Propionate |
| Glucocorticoid receptor | P04150 | Fluocinolone Acetonide |
| Glucocorticoid receptor | P04150 | Triamcinolone |
| Glucocorticoid receptor | P04150 | Prednisone |
| Glucocorticoid receptor | P04150 | Flumethasone |
| Glucocorticoid receptor | P04150 | Fludrocortisone |
| Glucocorticoid receptor | P04150 | Hydrocortisone |
| Glucocorticoid receptor | P04150 | Mometasone |
| Glucocorticoid receptor | P04150 | Clocortolone |
| Glucocorticoid receptor | P04150 | Flurandrenolide |
| Glucocorticoid receptor | P04150 | Prednisolone |
| Glucocorticoid receptor | P04150 | Loteprednol |
| Glucocorticoid receptor | P04150 | Rimexolone |
| Glucocorticoid receptor | P04150 | Methylprednisolone |
| Glucocorticoid receptor | P04150 | Clobetasol propionate |
| Glucocorticoid receptor | P04150 | Fluocinonide |
| Glucocorticoid receptor | P04150 | Prednicarbate |
| Glucocorticoid receptor | P04150 | Budesonide |
| Glucocorticoid receptor | P04150 | Dexamethasone |
| Glucocorticoid receptor | P04150 | Desonide |
| Glucocorticoid receptor | P04150 | Cortisone acetate |
| Glucocorticoid receptor | P04150 | Paramethasone |
| Glutathione S-transferase | Q8MU52 | Chloroquine |
| Glutathione S-transferase A2 | P09210 | Chloroquine |
| Group IIE secretory phospholipase A2 | Q9NZK7 | Nimesulide |
| Heat shock protein HSP 90-alpha | P07900 | Nedocromil |
| Hematopoietic prostaglandin D synthase | O60760 | Tranilast |
| High affinity immunoglobulin gamma Fc receptor I | P12314 | Etanercept |
| High affinity immunoglobulin gamma Fc receptor I | P12314 | Adalimumab |
| Histamine H1 receptor | P35367 | Olopatadine |
| Histamine H1 receptor | P35367 | Azelastine |
| Inhibitor of nuclear factor kappa-B kinase subunit alpha | O15111 | Mesalazine |
| Inhibitor of nuclear factor kappa-B kinase subunit alpha | O15111 | Sulfasalazine |
| Inhibitor of nuclear factor kappa-B kinase subunit beta | O14920 | Mesalazine |
| Inhibitor of nuclear factor kappa-B kinase subunit beta | O14920 | Sulfasalazine |
| Inhibitor of nuclear factor kappa-B kinase subunit beta | O14920 | Acetylsalicylic acid |
| Inosine-5'-monophosphate dehydrogenase 1 | P20839 | Mycophenolate mofetil |
| Inosine-5'-monophosphate dehydrogenase 1 | P20839 | Mycophenolic acid |
| Inosine-5'-monophosphate dehydrogenase 2 | P12268 | Mycophenolate mofetil |
| Inosine-5'-monophosphate dehydrogenase 2 | P12268 | Mycophenolic acid |
| Interferon gamma | P01579 | Olsalazine |
| Interferon gamma | P01579 | Apremilast |
| Interleukin-2 | P60568 | Apremilast |
| Lactotransferrin | P02788 | Nimesulide |
| Lactotransferrin | P02788 | Parecoxib |
| Lactoylglutathione lyase | Q04760 | Indomethacin |
| Low affinity immunoglobulin gamma Fc region receptor II-a | P12318 | Etanercept |
| Low affinity immunoglobulin gamma Fc region receptor II-a | P12318 | Adalimumab |
| Low affinity immunoglobulin gamma Fc region receptor II-b | P31994 | Etanercept |
| Low affinity immunoglobulin gamma Fc region receptor II-b | P31994 | Adalimumab |
| Low affinity immunoglobulin gamma Fc region receptor II-c | P31995 | Etanercept |
| Low affinity immunoglobulin gamma Fc region receptor II-c | P31995 | Adalimumab |
| Low affinity immunoglobulin gamma Fc region receptor III-A | P08637 | Etanercept |
| Low affinity immunoglobulin gamma Fc region receptor III-A | P08637 | Adalimumab |
| Low affinity immunoglobulin gamma Fc region receptor III-B | O75015 | Etanercept |
| Low affinity immunoglobulin gamma Fc region receptor III-B | O75015 | Adalimumab |
| Lymphotoxin-alpha | P01374 | Etanercept |
| Mineralocorticoid receptor | P08235 | Fluticasone Propionate |
| Mineralocorticoid receptor | P08235 | Fludrocortisone |
| Mitogen-activated protein kinase 3 | P27361 | Sulindac |
| Myeloperoxidase | P05164 | Mesalazine |
| NF-kappa-B inhibitor alpha | P25963 | Acetylsalicylic acid |
| Nitric oxide synthase, endothelial | P29474 | Apremilast |
| Nitric oxide synthase, inducible | P35228 | Dexamethasone |
| Nuclear factor NF-kappa-B p100 subunit | Q00653 | Acetylsalicylic acid |
| Nuclear factor NF-kappa-B p105 subunit | P19838 | Acetylsalicylic acid |
| Nuclear receptor subfamily 0 group B member 1 | P51843 | Dexamethasone |
| Peptidyl-prolyl cis-trans isomerase FKBP1A | P62942 | Pimecrolimus |
| Peptostreptococcal albumin-binding protein | Q51911 | Naproxen |
| Peroxisome proliferator-activated receptor alpha | Q07869 | Indomethacin |
| Peroxisome proliferator-activated receptor delta | Q03181 | Sulindac |
| Peroxisome proliferator-activated receptor gamma | P37231 | Mesalazine |
| Peroxisome proliferator-activated receptor gamma | P37231 | Indomethacin |
| Peroxisome proliferator-activated receptor gamma | P37231 | Sulfasalazine |
| Peroxisome proliferator-activated receptor gamma | P37231 | Balsalazide |
| Peroxisome proliferator-activated receptor gamma | P37231 | Ibuprofen |
| Phospholipase A2 | P04054 | Sulfasalazine |
| Phospholipase A2 | P04054 | Niflumic Acid |
| Phospholipase A2, membrane associated | P14555 | Indomethacin |
| Phospholipase A2, membrane associated | P14555 | Diclofenac |
| Potassium voltage-gated channel subfamily KQT member 2 | O43526 | Diclofenac |
| Potassium voltage-gated channel subfamily KQT member 2 | O43526 | Meclofenamic acid |
| Potassium voltage-gated channel subfamily KQT member 3 | O43525 | Diclofenac |
| Potassium voltage-gated channel subfamily KQT member 3 | O43525 | Meclofenamic acid |
| Progesterone receptor | P06401 | Fluticasone Propionate |
| Prostacyclin synthase | Q16647 | Phenylbutazone |
| Prostaglandin D2 receptor | Q13258 | Nedocromil |
| Prostaglandin D2 receptor 2 | Q9Y5Y4 | Indomethacin |
| Prostaglandin D2 receptor 2 | Q9Y5Y4 | Sulindac |
| Prostaglandin G/H synthase 1 | P23219 | Mesalazine |
| Prostaglandin G/H synthase 1 | P23219 | Indomethacin |
| Prostaglandin G/H synthase 1 | P23219 | Nabumetone |
| Prostaglandin G/H synthase 1 | P23219 | Ketorolac |
| Prostaglandin G/H synthase 1 | P23219 | Tenoxicam |
| Prostaglandin G/H synthase 1 | P23219 | Tolmetin |
| Prostaglandin G/H synthase 1 | P23219 | Piroxicam |
| Prostaglandin G/H synthase 1 | P23219 | Fenoprofen |
| Prostaglandin G/H synthase 1 | P23219 | Diclofenac |
| Prostaglandin G/H synthase 1 | P23219 | Sulindac |
| Prostaglandin G/H synthase 1 | P23219 | Flurbiprofen |
| Prostaglandin G/H synthase 1 | P23219 | Etodolac |
| Prostaglandin G/H synthase 1 | P23219 | Mefenamic acid |
| Prostaglandin G/H synthase 1 | P23219 | Naproxen |
| Prostaglandin G/H synthase 1 | P23219 | Sulfasalazine |
| Prostaglandin G/H synthase 1 | P23219 | Phenylbutazone |
| Prostaglandin G/H synthase 1 | P23219 | Meloxicam |
| Prostaglandin G/H synthase 1 | P23219 | Carprofen |
| Prostaglandin G/H synthase 1 | P23219 | Diflunisal |
| Prostaglandin G/H synthase 1 | P23219 | Suprofen |
| Prostaglandin G/H synthase 1 | P23219 | Salicylic acid |
| Prostaglandin G/H synthase 1 | P23219 | Meclofenamic acid |
| Prostaglandin G/H synthase 1 | P23219 | Acetylsalicylic acid |
| Prostaglandin G/H synthase 1 | P23219 | Bromfenac |
| Prostaglandin G/H synthase 1 | P23219 | Oxaprozin |
| Prostaglandin G/H synthase 1 | P23219 | Ketoprofen |
| Prostaglandin G/H synthase 1 | P23219 | Balsalazide |
| Prostaglandin G/H synthase 1 | P23219 | Ibuprofen |
| Prostaglandin G/H synthase 1 | P23219 | Lumiracoxib |
| Prostaglandin G/H synthase 1 | P23219 | Magnesium salicylate |
| Prostaglandin G/H synthase 1 | P23219 | Salsalate |
| Prostaglandin G/H synthase 1 | P23219 | Trisalicylate-choline |
| Prostaglandin G/H synthase 1 | P23219 | Antipyrine |
| Prostaglandin G/H synthase 1 | P23219 | Tiaprofenic acid |
| Prostaglandin G/H synthase 1 | P23219 | Niflumic Acid |
| Prostaglandin G/H synthase 1 | P23219 | Lornoxicam |
| Prostaglandin G/H synthase 1 | P23219 | Nepafenac |
| Prostaglandin G/H synthase 2 | P35354 | Mesalazine |
| Prostaglandin G/H synthase 2 | P35354 | Indomethacin |
| Prostaglandin G/H synthase 2 | P35354 | Nabumetone |
| Prostaglandin G/H synthase 2 | P35354 | Ketorolac |
| Prostaglandin G/H synthase 2 | P35354 | Tenoxicam |
| Prostaglandin G/H synthase 2 | P35354 | Celecoxib |
| Prostaglandin G/H synthase 2 | P35354 | Tolmetin |
| Prostaglandin G/H synthase 2 | P35354 | Piroxicam |
| Prostaglandin G/H synthase 2 | P35354 | Fenoprofen |
| Prostaglandin G/H synthase 2 | P35354 | Diclofenac |
| Prostaglandin G/H synthase 2 | P35354 | Sulindac |
| Prostaglandin G/H synthase 2 | P35354 | Flurbiprofen |
| Prostaglandin G/H synthase 2 | P35354 | Etodolac |
| Prostaglandin G/H synthase 2 | P35354 | Mefenamic acid |
| Prostaglandin G/H synthase 2 | P35354 | Naproxen |
| Prostaglandin G/H synthase 2 | P35354 | Sulfasalazine |
| Prostaglandin G/H synthase 2 | P35354 | Phenylbutazone |
| Prostaglandin G/H synthase 2 | P35354 | Meloxicam |
| Prostaglandin G/H synthase 2 | P35354 | Carprofen |
| Prostaglandin G/H synthase 2 | P35354 | Diflunisal |
| Prostaglandin G/H synthase 2 | P35354 | Suprofen |
| Prostaglandin G/H synthase 2 | P35354 | Salicylic acid |
| Prostaglandin G/H synthase 2 | P35354 | Meclofenamic acid |
| Prostaglandin G/H synthase 2 | P35354 | Acetylsalicylic acid |
| Prostaglandin G/H synthase 2 | P35354 | Bromfenac |
| Prostaglandin G/H synthase 2 | P35354 | Oxaprozin |
| Prostaglandin G/H synthase 2 | P35354 | Ketoprofen |
| Prostaglandin G/H synthase 2 | P35354 | Balsalazide |
| Prostaglandin G/H synthase 2 | P35354 | Ibuprofen |
| Prostaglandin G/H synthase 2 | P35354 | Lumiracoxib |
| Prostaglandin G/H synthase 2 | P35354 | Magnesium salicylate |
| Prostaglandin G/H synthase 2 | P35354 | Salsalate |
| Prostaglandin G/H synthase 2 | P35354 | Trisalicylate-choline |
| Prostaglandin G/H synthase 2 | P35354 | Antipyrine |
| Prostaglandin G/H synthase 2 | P35354 | Tiaprofenic acid |
| Prostaglandin G/H synthase 2 | P35354 | Etoricoxib |
| Prostaglandin G/H synthase 2 | P35354 | Niflumic Acid |
| Prostaglandin G/H synthase 2 | P35354 | Nimesulide |
| Prostaglandin G/H synthase 2 | P35354 | Lornoxicam |
| Prostaglandin G/H synthase 2 | P35354 | Nepafenac |
| Prostaglandin G/H synthase 2 | P35354 | Parecoxib |
| Prostaglandin reductase 2 | Q8N8N7 | Indomethacin |
| Protein S100-A1 | P23297 | Olopatadine |
| Protein S100-A12 | P80511 | Olopatadine |
| Protein S100-A13 | Q99584 | Olopatadine |
| Protein S100-A2 | P29034 | Olopatadine |
| Protein S100-B | P04271 | Olopatadine |
| Protein-tyrosine kinase 2-beta | Q14289 | Leflunomide |
| Retinoic acid receptor alpha | P10276 | Adapalene |
| Retinoic acid receptor beta | P10826 | Adapalene |
| Retinoic acid receptor gamma | P13631 | Adapalene |
| Retinoic acid receptor RXR-alpha | P19793 | Adapalene |
| Retinoic acid receptor RXR-alpha | P19793 | Etodolac |
| Retinoic acid receptor RXR-beta | P28702 | Adapalene |
| Retinoic acid receptor RXR-gamma | P48443 | Adapalene |
| Ribosomal protein S6 kinase alpha-3 | P51812 | Acetylsalicylic acid |
| Serine/threonine-protein kinase mTOR | P42345 | Pimecrolimus |
| Smoothened homolog | Q99835 | Fluocinonide |
| Smoothened homolog | Q99835 | Halcinonide |
| Sodium channel protein type 4 subunit alpha | P35499 | Diclofenac |
| Squalene monooxygenase | Q14534 | Naftifine |
| Thiopurine S-methyltransferase | P51580 | Olsalazine |
| Thrombomodulin | P07204 | Ibuprofen |
| Thromboxane-A synthase | P24557 | Sulfasalazine |
| Tissue-type plasminogen activator | P00750 | Ibuprofen |
| Toll-like receptor 9 | Q9NR96 | Chloroquine |
| Tumor necrosis factor | P01375 | Etanercept |
| Tumor necrosis factor | P01375 | Adalimumab |
| Tumor necrosis factor | P01375 | Chloroquine |
| Tumor necrosis factor | P01375 | Apremilast |
| Tumor necrosis factor receptor superfamily member 1B | P20333 | Etanercept |
| UDP-glucuronosyltransferase 1-9 | O60656 | Niflumic Acid |
| Uncharacterized oxidoreductase CzcO-like | Q5L2G3 | Clofazimine |

**Additional file 3 Introduction of experimental models in AICD**

MS_001

Cell based gene expression assay

An assay evaluates the expression level of a protein target in cells.

MS_002

Cell proliferation assay

An assay detects the number of cells after treatment with certain stimuli.

MS_003

Cell viability assay

An assay distinguishes viable or dead cells after treatment with certain stimuli.

MS_004

Competitive activity assay

An assay evaluates the molecular function of a protein target in the presence of known ligand and testing chemical candidate.

MS_005

Competitive binding assay

An assay evaluates the binding ability of a chemical molecule to a protein target (usually a receptor) in the presence of known ligand.

MS_006

Enzyme activity assay

An assay is developed based on the molecular function of enzymes. The chemical molecule was considered as a potential enzyme inhibitor when it blocks enzymatic reaction.

MS_007

Ion channel activity assay (Calcium assay)

An assay evaluates the activity of an ion channel by monitoring the influx of calcium.

MS_008

Kinase activity assay

An assay evaluates the activity of a kinase by detecting the phosphorylation level of kinase substrate.

MS_009

Receptor activity assay (cAMP assay)

An assay evaluates a receptor’s activity (the protein target) by detecting intracellular level of cAMP.

MS_010

Receptor activity assay

An assay evaluates a receptor’s activity by detecting its downstream molecular events. In some cases, this assay detects gene expression, kinase activation or metabolites which may be induced by the receptor.

MS_011

Receptor activity assay (Calcium assay)

An assay evaluates the activity of a receptor by monitoring the influx of calcium.

MS_012

Receptor activity assay (chemotaxis assay)

An assay evaluates the activity of a receptor by detecting the cell migration ability.

MS_013

Receptor kinase activity assay

An assay evaluates the activity of a receptor kinase by detecting the phosphorylation level of its substrate.

MS_014

Nuclear translocation assay

An assay evaluates the subcellular location (cell nucleus) of a protein target in response to certain stimuli.

MS_015

Transcriptional activity assay

An assay evaluates the activity of a transcription factor by detecting the expression level of a gene (usually Luciferase) which is driven by a promoter containing its binding sites.

MS_016

in vivo assay

An assay evaluates the molecular function of a protein target on the level of living animals (behavioral assay) or tissues (biochemistry assay).

**Supplementary Dataset 1: The final list of the small-molecules and targets along with their collected chemical/biological properties.xlsx**

**Supplementary Dataset 2: Degree values of molecules and targets in AICD.xlsx**

**Supplementary Dataset 3: Supplementary_materials_Reactome.xlsx**

**Supplementary Dataset 4: Supplementary_materials_BP.xlsx**

**Supplementary Dataset 5: Supplementary_materials_Diseases.xlsx**

**Supplementary Dataset 6: The network data of all molecules and targets.xlsx**

**Supplementary Dataset 7: The network of all molecules and targets.zip**

**Supplementary Dataset 8: The network data of molecules and targets (Degree≥2）.xlsx**

**Supplementary Dataset 9: The network of molecules and targets (Degree≥2）.zip**
